# Bacterial Lipopolysaccharide Increases Serotonin Metabolism in Both Medial Prefrontal Cortex and Nucleus Accumbens in Male Wild Type Rats, but Not in Serotonin Transporter Knockout Rats

**DOI:** 10.3390/ph11030066

**Published:** 2018-07-05

**Authors:** Gerdien A. H. Korte-Bouws, Floor van Heesch, Koen G. C. Westphal, Lisa M. J. Ankersmit, Edwin M. van Oosten, Onur Güntürkün, S. Mechiel Korte

**Affiliations:** 1 Division of Pharmacology, Utrecht Institute for Pharmaceutical Sciences (UIPS), Utrecht University, Faculty of Science, Universiteitsweg 99, 3584 CG Utrecht, The Netherlands; floorvheesch@hotmail.com (F.v.H.); K.G.C.Westphal@uu.nl (K.G.C.W.); l.m.j.ankersmit@students.uu.nl (L.M.J.A.); e.m.vanoosten@students.uu.nl (E.M.v.O.); s.m.korte@uu.nl (S.M.K.); 2 Department of Biopsychology, Faculty of Psychology, Ruhr-Universität Bochum, Universitätsstraße 150, D-44780 Bochum, Germany; onur.guentuerkuen@ruhr-uni-bochum.de

**Keywords:** lipopolysaccharide, proinflammatory cytokines, serotonin transporter, Slc6a41, metabolism, microdialysis, medial prefrontal cortex, nucleus accumbens

## Abstract

It is well known that bacterial lipopolysaccharides (LPS) both increases proinflammatory cytokines and produces sickness behavior, including fatigue and anhedonia (i.e., the inability to experience pleasure). Previously, we have shown that intraperitoneally (i.p.) administered LPS increased extracellular monoamine metabolite levels in the nucleus accumbens (NAc) and medial prefrontal cortex (mPFC), which was completely, or at least partly, prevented by pretreatment with a triple reuptake inhibitor that also blocks the serotonin (5-HT) transporter (SERT). This suggests indirectly, that LPS may enhance SERT transporter activity, and consequently, increase removal of 5-HT from the synaptic cleft, and increase metabolism of 5-HT. In the present study, we focus more specifically on the role of SERT in this increased metabolism by using rats, that differ in SERT expression. Therefore, the effects of an intraperitoneal LPS injection on extracellular concentrations of 5-HT and its metabolite 5-hydroxyindoleacetic acid (5-HIAA) were investigated by in vivo microdialysis in the NAc and mPFC of wild type (SERT^+/+^), heterozygous (SERT^+/−^) and knockout (SERT^−/−^) rats. Here, we show that LPS-induced 5-HIAA formation in male rats, is significantly increased in SERT^+/+^ rats in both the NAc and mPFC, whereas this increase is partly or totally abolished in SERT^+/−^ and SERT^−/−^ rats, respectively. Thus, the present study supports the hypothesis that systemic LPS in male rats increases SERT function and consequently enhances 5-HT uptake and metabolism in both the NAc and mPFC.

## 1. Introduction

An increasing amount of evidence suggests that alterations in proinflammatory cytokines play an important role in major depression, particularly in depression due to a medical condition [1,2,3,4]. For example, patients with chronic inflammatory diseases, such as rheumatoid arthritis, inflammatory bowel disease or psoriasis, have an increased risk of developing sickness behavior, which has a large overlap with depression-like symptoms, such as fatigue, disturbed cognition and anhedonia [5,6,7,8].

Anhedonia, i.e., the inability to experience pleasure, is a core symptom of major depression that can be assessed in rodents with the intracranial self-stimulation procedure [9]. Previously, it has been reported that systemic bacterial lipopolysaccharides (LPS) strongly activates the immune system to release proinflammatory cytokines, that induces anhedonia in rats [10,11,12]. Interestingly, previously we have shown that LPS-induced anhedonia is not observed in serotonin (5-HT) transporter knockout (SERT^−/−^) rats, while LPS-induced anhedonia was decreased in heterozygous rats (SERT^+/−^) compared to wild type animals (SERT^+/+^) [11]. As was previously reported, SERT is completely absent in SERT knockout rats, while heterozygous rats have been shown to have 48–80% SERT activity compared to wild type animals [13]. This indirectly suggests that absence of SERT protects the animals from LPS-induced anhedonia.

The exact mechanism why SERT knockout rats are not susceptible to LPS-induced anhedonia is still unknown. However, since selective serotonin reuptake inhibitors (SSRIs) are expected to alleviate depression by increasing 5-HT availability through inhibition of SERT, differences in 5-HT availability could be responsible for the resistance to LPS-induced anhedonia in SERT knockout rats [14].

Previously, we have shown that i.p. administered LPS increased extracellular 5-HT metabolite (5-HIAA) levels in the nucleus accumbens (NAc) and medial prefrontal cortex (mPFC), which was, completely or at least partly, prevented by pre-treatment with the reuptake inhibitor DOV 216,303, that also blocks SERT. This suggests indirectly, that LPS-induced increased SERT activity does not only lead to increasing removal of 5-HT from the synaptic cleft, but it consequently also leads to increase metabolism of 5-HT.

Therefore, specifically the role of SERT was examined by using heterozygous and knockout SERT male rats. In the present microdialysis experiments, systemic LPS was administered to study serotonergic metabolism (i.e., breakdown of 5-HT to 5-HIAA) in the NAc and mPFC of SERT^+/−^, and ^SERT−/−^ male rats in comparison to SERT^+/+^ male rats.

## 2. Results

### 2.1. Microdialysis

#### 2.1.1. The Effect of Peripheral LPS on 5-HT and 5-HIAA Levels in the NAc and mPFC of Wild Type Rats and SERT (Partial and Total) Knockout Rats

**Figure 1:**
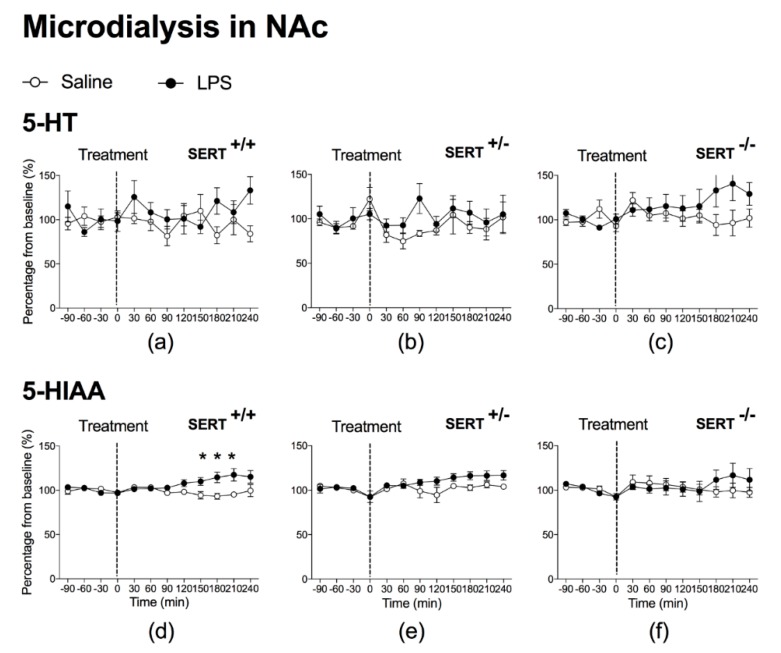
Microdialysis in the Nucleus Accumbens (NAc). At *t* = 0 min, rats received Saline or lipopolysaccharides (LPS). Graph represents percentage from baseline (%) of 5-HT and 5-HIAA from each time point (min). Animals (*n*) per group were as follows: *n* = 6 for Saline-SERT^+/+^ rats (**a**,**d**) and n = 7 for LPS-SERT^+/+^ rats (**a**,**d**); *n* = 3 for Saline-SERT^+/−^ rats (**b**,**e**) and *n* = 7 for LPS-SERT^+/−^ rats (**b**,**e**); *n* = 5 for Saline-SERT^−/−^ rats (**c**,**f**) and *n* = 5 for LPS-SERT^−/−^ rats (**c**,**f**). Significance: * *p* < 0.05.

**Figure 2:**
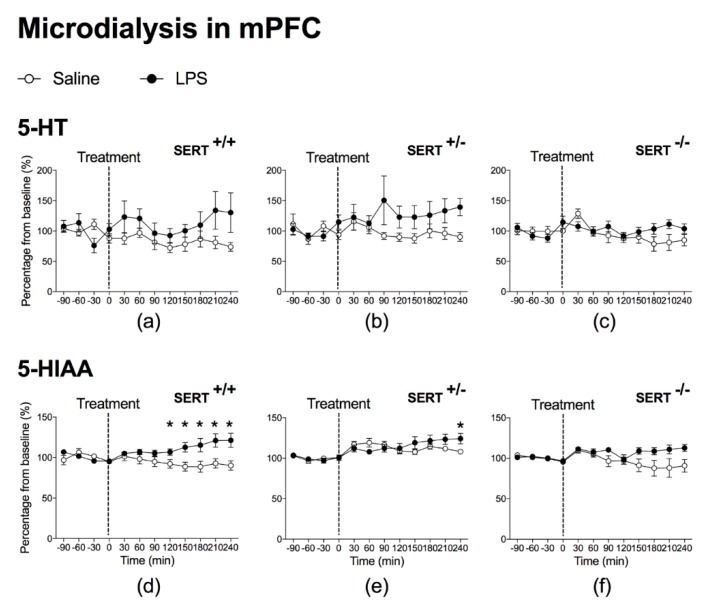
Microdialysis in the medial Prefrontal Cortex (mPFC). At *t* = 0 min, rats received Saline or LPS. Graph represents percentage from baseline (%) of 5-HT and 5-HIAA from each time point (min). Animals (*n*) per group were as follows: *n* = 7 for Saline-SERT^+/+^ rats (**a**,**d**) and *n* = 7 for LPS-SERT^+/+^ rats (**a**,**d**); *n* = 7 for Saline-SERT^+/−^ rats (**b**,**e**) and *n* = 8 for LPS-SERT^+/−^ rats (**b**,**e**); *n* = 8 for Saline-SERT^−/−^ rats (**c**,**f**) and *n* = 7 for LPS-SERT^−/−^ rats (**c**,**f**). Significance: * *p* < 0.05.

LPS did not produce significant overall effects on 5-HT levels in the NAc and mPFC of SERT^+/+^ (Figure 1a and Figure 2a, respectively), SERT^+/−^ rats (Figure 1b and Figure 2b, respectively) and SERT^−/−^ rats (Figure 1c and Figure 2c, respectively). Neither did post hoc analyses reveal a significant difference at any time point.

For the serotonergic metabolite 5-HIAA in the NAc of SERT^+/+^ rats, however, significant LPS effects were found, as indicated by a significant time × treatment interaction: F(1.8,20.0) = 6.0, *p* = 0.011, ε = 0.260 (Figure 1d), while the overall LPS treatment effect was almost significant: F(1.0,11) = 4.8, *p* = 0.051, ε = 0.260. Post hoc *t*-tests showed that accumbal 5-HIAA levels increased significantly at 150 min, 180 min and 210 min after exposure to LPS (Figure 1d).

LPS also increased 5-HIAA levels in the mPFC of SERT^+/+^ rats, as indicated by an overall treatment effect: F(1.0,12) = 6.2, *p* = 0.029, ε = 0.281, together with a time × treatment interaction: F(2.0,23.6) = 6.3, *p* = 0.007, ε = 0.281 (Figure 2d). Post hoc *t*-tests showed that 5-HIAA levels in the mPFC were significantly increased at 120 min, 150 min, 180 min, 210 min and 240 min after exposure to LPS (Figure 2d). LPS induced a significant small increase in 5-HIAA levels in mPFC of SERT^+/−^ rats, as indicated by a significant time × treatment interaction (F(3.0,39.3) = 5.7, *p* = 0.002, ε = 0.432 (Figure 2e)), although there was no overall LPS treatment effect. Post hoc analysis revealed that 5-HIAA levels in the mPFC were significantly increased at 240 min after exposure to LPS. In contrast, LPS did not significantly affect 5-HIAA levels in SERT^−/−^ rats at any time point, neither in the mPFC nor in the NAc. As a consequence, neither time × treatment interactions in the NAc (Figure 1f) and in the mPFC were found (Figure 2f), nor a significant effect at any time point.

#### 2.1.2. Baseline 5-HT and 5-HIAA Levels in the NAc and mPFC of Wild Type Rats and SERT (Partial and Total) Knockout Rats

Under the baseline conditions, 5-HT levels in the NAc, as well as in the mPFC, were significantly higher in SERT^−/−^ rats compared to SERT^+/+^ (NAc: *p* < 0.001 and mPFC: *p* < 0.001) and SERT^+/−^ rats (NAc: *p* < 0.001 and mPFC: *p* < 0.001) (see Table 1). Remarkably, SERT^+/+^ and SERT^+/−^ rats did not differ from each other in respect to extracellular 5-HT levels in both brain areas. Also, extracellular 5-HIAA levels differed significantly between genotypes in both brain structures. As expected, in the NAc as well as in the mPFC, SERT^−/−^ rats had significantly lower extracellular 5-HIAA levels as compared to SERT^+/+^ (NAc: *p* < 0.001 and mPFC: *p* < 0.001) and SERT^+/−^ rats (NAc: *p* = 0.008 and mPFC: *p* < 0.001). Furthermore, extracellular 5-HIAA level of SERT^+/−^ rats was significantly lower than 5-HIAA levels of SERT^+/+^ rats in both brain areas (NAc: *p* = 0.016 and mPFC: *p* = 0.001).

## 3. Discussion

The present microdialysis study shows that LPS-induced 5-HT metabolism (i.e., 5-HIAA formation) is significantly increased in both the NAc and mPFC of homozygous (SERT^+/+^) male rats, whereas this increase is partly or completely abolished in heterozygous (SERT^+/−^) or knockout (SERT^−/−^) male rats, respectively. These results support the hypothesis that systemic LPS administration increases SERT activity in CNS of male rats (possibly in neurons and/or astrocytes located in raphe nuclei, NAc or mPFC).

### 3.1. LPS Increases SERT Activity in Both NAc and mPFC

The observed LPS-induced increase in extracellular 5-HIAA levels in the mPFC and NAc of normal (SERT^+/+^) rats is in agreement with in vitro, ex vivo and in vivo studies, showing that LPS increases SERT activity in neurons and thereby the reuptake of 5-HT, enabling more metabolism of 5-HT into 5-HIAA [10,15,16,17,18,19,20,21,22,23,24]. The partly or completely abolished LPS-induced increase in 5-HIAA in heterozygous (SERT^+/−^) or knockout (SERT^−/−^) rats, suggests a specific role of SERT in this process.

The somewhat larger effect of LPS on serotonergic activity in the mPFC may be explained by the fact that the mPFC is highly innervated by 5-HT neurons, whereas the NAc is not [25].

Previously, different research groups reported that LPS treatment increased both 5-HT release and 5-HIAA levels in hippocampus [26,27,28] and preoptic area, whereas only 5-HIAA was increased in the NAc [28]. In the present study, LPS did not significantly increase 5-HT in mPFC or NAc in male SERT^+/+^. Whether this is a discrepancy to these previous studies, or because it is the consequence of the measurement of 5-HT in different brain areas, needs more investigation.

The present data support the hypothesis that LPS enhances SERT activity, and thereby the reuptake of 5-HT. However, this is not reflected in a decrease of extracellular 5-HT concentrations below its baseline. Since SERT activity is neurotransmitter concentration dependent, it could be speculated that there is less reuptake of 5-HT when extracellular concentrations are below a certain baseline concentration. However, it is speculated, in contrast to acute infection, that chronic inflammation due to a lower set point is able to shift the baseline to lower concentrations. Therefore, at this moment, we are studying the effects of SERT activity and 5-HT reuptake in an animal model of chronic inflammation.

### 3.2. Underlying Mechanisms of Increased SERT Activity

**Figure 3:**
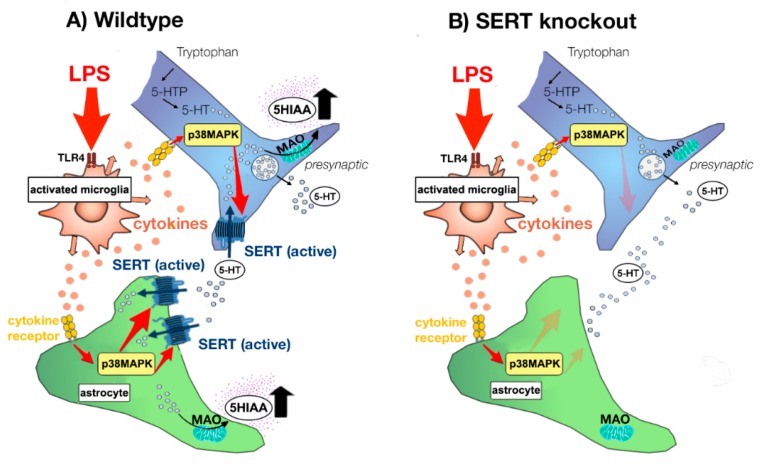
Model of regulation of serotonin (5-HT) transporter (SERT) activity after LPS challenge in SERT Wildtype (SERT^+/+^) rats and SERT Knockout (SERT^−/−^) rats. LPS via the binding to toll-like receptor 4 (TLR-4) on microglia produce its’ activation and consequently increase in the release of pro-inflammatory cytokines (e.g., IL-1, IL-6 and TNF-α). This increase in pro-inflammatory cytokines enhance SERT activity in neurons, as well as increase SERT gene expression in astrocytes. Both processes may be mediated by p38 MAPK-dependent pathways, despite the fact that also independent pathways have been suggested. After reuptake by SERT, 5-HT is packed in storage vesicles or metabolized by monoamine oxidase (MAO). In serotonergic neurons and astrocytes, MAO metabolizes 5-HT to 5-hydroxyindole acetaldehyde and thereafter aldehyde dehydrogenase rapidly metabolizes 5-hydroxyindole acetaldehyde to 5-hydroxyindolacetic acid (5-HIAA). Thereafter, 5-HIAA diffuses outside the cells. Thus, SERT plays, besides controlling the length of the cellular actions of 5-HT, an important role in the formation of its’ metabolite 5-HIAA. As a consequence, LPS-induced SERT activity results in enhanced 5-HT uptake into astrocytes and neurons and thereby promote 5-HT degradation to 5-HIAA in wildtype animals (**A**). Since SERT does not come to expression in SERT knockout animals, LPS cannot increase SERT activity and associated 5-HT metabolism in these animals (**B**), indicating that the SERT is involved in LPS-induced enhancement of 5-HT metabolism in male rats. (Figure adapted after Haase and Brown, 2015 [33]).

It is a known fact that LPS binds to toll-like receptor 4 (TLR4) on macrophages, astrocytes, and microglia to produce proinflammatory cytokines, such as IL-1, IL-6 and TNF-α [12,29,30]. It has been demonstrated that both LPS and proinflammatory cytokines increase monoamine transporter trafficking and function [16,24,31,32,33] (see Figure 3). There is growing body of evidence that this process is p38 MAPK-dependent [16,17,34,35,36,37]. Although, also other mechanisms than p38 MAPK have been proposed in the PFC [24]. In addition, other kinases may be associated with SERT regulation, such as Protein Kinase C, ERK1/2, phosphatidylinositol 3-Kinase/Akt and adenosine [31,34,38].

### 3.3. Baseline Differences in 5-HT and 5-HIAA Levels

Under baseline conditions (see Table 1), we and others have found that extracellular 5-HT levels are significantly increased in hippocampus, NAc and mPFC of SERT^−/−^ rats compared to SERT^+/+^ and/or SERT^+/−^ rats and mice [13,39,40], whereas extracellular levels of 5-HIAA are significantly decreased [34]. These results can be explained by the fact that due to the absence of 5-HT transporters in SERT^−/−^ rats, there is no reuptake of 5-HT into the serotonergic neuron, and consequently no breakdown of 5-HT into 5-HIAA by MAO (monoamine oxidase). This explains why SERT^−/−^ rats have lower 5-HIAA levels and higher levels of 5-HT in the extracellular space.

Although SERT activity is completely absent in SERT^−/−^ rats, limited formation of 5-HIAA is still present, possibly because alternative routes can lead to reuptake of 5-HT and corresponding 5-HIAA formation as well. Indeed, it has been demonstrated that the maximum rate (Vmax) of 5-HT uptake in the hippocampus is reduced by 13.4% in SERT^+/−^ rats and by 72.2% in SERT^−/−^ rats [13]. Interestingly, inhibition of noradrenaline transporters (NET), but not dopamine transporters (DAT), attenuated the reuptake of the remaining 5-HT in the hippocampus of SERT^−/−^ rats [13,38]. Furthermore, dopaminergic neurons in the ventral tegmental area and substantia nigra have been shown to take up 5-HT by DAT in SERT^−/−^ mice [41]. Moreover, it has been demonstrated that NET and DAT concentrations were not different from genotypes throughout the brain [13].

Previously, it has been shown that SERT wild type male rats and SERT knockout male rats do not differ in tryptophan levels, tryptophan (TRP)/total long neutral amino acids (∑LNAA) ratios, tryptophan hydroxylase activity and MAO-A activity [13,42]. In addition, in mice, baseline tryptophan levels were not different between SERT wild type and SERT knockout animals, but tryptophan levels were shown to be higher in female mice as compared to male mice [23]. Furthermore, it has been shown that LPS treatment caused an increase in brain tryptophan levels [22]. In addition, serotonin synthesis is increased in SERT knockout mice, in particular in females [23].

Because the NAc is a smaller brain region than the mPFC in which to insert a microdialysis probe, a probe length of 2 mm was used in the NAc, in contrast to a 3 mm probe length in the mPFC. This may partly explain the higher baseline neurotransmitter concentrations in the mPFC as compared to NAc, although it cannot be excluded that a possibly lower SERT expression in the mPFC than in the NAc, plays a role too [43], which is in agreement with the higher 5-HIAA levels in NAc as compared to mPFC (see Table 1).

### 3.4. Consequences of LPS- and Cytokine-Induced Increases in SERT Activity

There are strong indications that LPS- and proinflammatory cytokine-induced increase in SERT function are necessary for the development of depression-like behavior, including fatigue and anhedonia.

Further, indications for an important role for increased SERT activity in LPS-induced anhedonia arises from clinical research showing that LPS-induced depressive symptoms could be reduced by 5-day pre-treatment with the SSRI citalopram [44]. Besides, prophylactic administration of SSRIs can be successfully used in a subgroup of patients who are at risk of developing major depression during IFN-α treatment [45]. Previously, it was shown that LPS-induced depression-like behavior (despair and anhedonia, i.e., the inability to experience pleasure probably caused by reduced ability to experience reward), as measured in the tail-suspension test [17] and in the intracranial self-stimulation (ICSS) paradigm, respectively, was abolished in knockout (SERT^−/−^) animals [10,11,46].

In addition, it has been shown in mice, albeit not in rats, that the midline raphe nuclei express in IL-1 receptors [47] and that IL-1β- and LPS-induced increase SERT activity and LPS-induced immobility in the tail suspension test, a measure of behavioral despair, were abolished in IL-1R knockout mice [17]. Moreover, inhibition of the p38 MAPK signaling pathway with SB203580 blocked IL-1β-induced stimulation of SERT [16]. In addition, the IL-1 signaling pathway is pivotally involved in the development of LPS-induced anxiolytic-like behavior in elevated-plus maze test and immobility behavior in both forced-swim test and tail suspension test via a p38 MAPK-dependent pathway in serotonergic dorsal raphe neurons [48], whereas p38 MAPK does not seem to be involved in increased SERT function in the PFC [24].

Interestingly, mice that are more susceptible to develop anhedonia have increased microglial activation, increased TNF-α and SERT expression in the PFC [49].

Remarkably, sex differences in compensatory mechanisms have been reported in SERT knockout mice [43]. Therefore, in the future, more experiments are needed to investigate to what extent sex differences play a role in the suggested mechanisms.

## 4. Materials and Methods

### 4.1. Animals

Male serotonin transporter (Slc6a41Hubr) knockout rats generated by *N*-ethyl-*N*-nitrosourea (ENU)-induced mutagenesis [50] were bred and reared in the animal facilities of the Utrecht University. Animals were bred by crossing serotonin transporter heterozygous rats (SERT^+/−^). At the age of 21 days, pups were weaned and ear cuts were taken for genotyping. Animals were placed on a 12 h light-dark cycle with lights on at 6:00 a.m. and of at 6:00 p.m. Food and water were available ad libitum. Animals were housed 4 per cage. Each animal was housed with litter mates having the same genotype (SERT^+/+^, SERT^+/−^ or SERT^−/−^) and undergoing the same treatment (saline or LPS). Experiments started once animals were weighing 290–350 g. The study was conducted in accordance with the European and Dutch governmental guidelines and approved by the Ethical Committee for Animal Research of Utrecht University, The Netherlands (license no. DEC 2011.I.01.005).

### 4.2. Drugs

*E. coli* derived lipopolysaccharide (LPS) (art no. 0127:B8 Sigma-Aldrich Chemie N.V., Zwijndrecht, The Netherlands) was dissolved in saline (0.9% NaCl in demi water) and prepared freshly on test days from the stock solution (0.5 mg/mL dissolved in saline). LPS (250 µg/kg) was administered intraperitoneally (i.p.) in a volume of 2 mL/kg. Control animals received i.p. injections of saline in a volume of 2 mL/kg.

### 4.3. Microdialysis

#### 4.3.1. Microdialysis Surgery

All rats (*n* = 48) were anesthetized by inhalation of a mixture of isoflurane gas (2%) and oxygen and were placed in a stereotaxic instrument. Two cuprophane microdialysis probes were implanted per rat. One probe was implanted in the NAc, the other in the mPFC (MAB 4.6.2 CU and MAB 4.7.3 CU, for NAc and mPFC, respectively (Microbiotech/se AB, Stockholm, Sweden)). The coordinates of the NAc and mPFC were anteroposterior +1.6 mm; mediolateral +1.8 mm (under a 0° angle) from bregma; dorsoventral −8.2 mm from skull surface and anteroposterior +3.2 mm; mediolateral +1.0 mm (under a 0° angle) from bregma; dorsoventral −4.0 mm from skull surface, respectively [51]. Probes were anchored with non-acrylic dental cement on the skull. After implantation of the microdialysis probes, rats were housed individually and placed in the microdialysis room until the end of the experiment.

#### 4.3.2. Microdialysis Experiment

The microdialysis experiment was performed in conscious freely moving rats, one day after implantation of the microdialysis probes. A pump (KdScientific Pump 220 series, KD Scientific Inc., Holliston, MA, USA) perfused the system with Ringer solution (147 mM NaCl, 2.3 mM KCl, 2.3 mM CaCl_2_ and 1 mM MgCl_2_) at a constant flow rate of 0.02 mL/h. During microdialysis, the flow rate was set at 0.09 mL/h. At 8:00 a.m. rats were connected to a dual channel swivel (type 375/D/22QM, Microbiotech) which allowed them to move freely. Three hours after connection, samples were manually collected every 30 min in vials containing 15 µL of 0.1 M acetic acid and frozen at −80 °C until analysis with HPLC. From 11:00 a.m. until 1:00 p.m. four baseline samples were collected. Subsequently, the animals were injected i.p. with saline (SERT^+/+^ *n* = 8; SERT^+/−^ *n* = 8 and SERT^−/−^ *n* = 8) or LPS (SERT^+/+^ *n* = 8; SERT^+/−^ *n* = 8 and SERT^−/−^ *n* = 8). During the whole experiment, 12 samples were collected per rat. At the end of the microdialysis experiment all animals were sacrificed immediately. The brains were dissected and stored in formaldehyde 30% to verify probe localization later on.

#### 4.3.3. HPLC

Microdialysis samples were stored at −80 °C until analysis. 5-HT and its metabolite 5-HIAA were detected simultaneously by HPLC with electrochemical detection using an Alexys 100 LC-EC system (Antec Scientific, Zoeterwoude, The Netherlands). The system consisted of two pumps, one auto sampler with a 10-port injection valve, two columns and two detector cells. Column 1 (NeuroSep105 C18 1 × 50 mm, 3 μm particle size) in combination with detector cell 1, separated and detected 5-HT, whereas column 2 (NeuroSep 115 C18 1 × 150 mm, 3 μm particle size) in combination with detector cell 2, separated and detected 5-HIAA. The mobile phase for column 1 consisted of 50 mM phosphoric acid, 8 mM KCl, 0.1 mM EDTA (pH 6.0), 18.5 % methanol and 400 mg/L OSA. The mobile phase for column 2 consisted of 50 mM phosphoric acid, 50 mM citric acid, 8 mM KCl, 0.1 mM EDTA (pH 3.25), 19.5 % methanol and 700 mg/L OSA. Both mobile phases were pumped at 50 µL/min. Samples were kept at 8 °C during analysis. From each microdialysis sample 5 μL was injected simultaneously onto each column. 5-HT and 5-HIAA were detected electrochemically using μVT-03 flow cells (Antec) with glassy carbon working electrodes. Potential settings were for 5-HT + 0.30 V versus Ag/AgCl and for 5-HIAA + 0.59 V versus Ag/AgCl. The columns and detector cells were kept at 35 °C in a column oven. The chromatogram was recorded and analyzed using the Alexys data system (Antec). The limit of detection was 0.05 nM (S/N ratio 3:1).

#### 4.3.4. Histology

**Figure 4:**
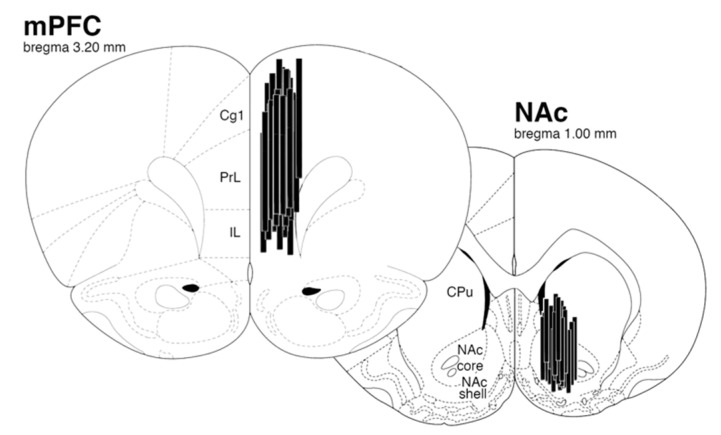
Localization of microdialysis probes in mPFC (3 mm length) and NAc (2 mm length). Cg1 = cingulate cortex, area 1; PrL = prelimbic cortex; IL = infralimbic cortex; CPu = caudate putamen; NAc core and shell = nucleus accumbens core and nucleus accumbens shell.

Two days before brain slicing, the brains were passed from formaldehyde to a 30% sucrose solution. Probe placements were verified on 60 µm cresyl violet stained sections that were cut on a cryostat (Leica CM3050, Leica Biosystems Inc., Buffalo Grove, IL, USA). Data were discarded when the microdialysis probe was outside the NAc or mPFC. In Figure 4, the correct probe localizations are shown of which data were used.

Some data could not be used due to obstruction of the microdialysis probe, or breaking of the outlet of the probe due to scratching and grooming from seven rats of the NAc study and from three rats of the mPFC study. Wrong probe localization caused eight dropouts in the NAc group, while this caused only one dropout in the mPFC group.

### 4.4. Statistics

Mean NAc and mPFC baseline 5-HT and 5-HIAA levels in SERT^+/+^, SERT^+/−^ and SERT^−/−^ rats were analyzed with use of one-way ANOVA. For each genotype, microdialysis measurements were expressed as a percentage of baseline and analyzed by repeated measures ANOVA with time (4 levels: −90 min, −60 min, −30 min and 0 min) as within subject factor and treatment (saline or LPS) as between subject factor to exclude differences between the saline and LPS groups at baseline. Subsequently, post-injection data was compared in a repeated measures ANOVA with time (8 levels: 30 min, 60 min, 90 min, 120 min, 150 min, 180 min, 210 min and 240 min) as within subject factor and treatment (saline or LPS) as between subject factor. In case of a significant time × treatment interaction, effects of LPS on individual time points were analyzed with post hoc *t*-tests with treatment (saline or LPS) as the grouping variable. When the assumption of sphericity was violated, the results were corrected by the Greenhouse-Geisser procedure. Threshold for significance level was set at *p* < 0.05.

## 5. Conclusions

The present study supports the hypothesis that systemic LPS administration increases 5-HT metabolism by increasing SERT function in the CNS of male rats, as reflected by higher 5-HIAA levels in both the NAc and mPFC, which were reduced or fully absent in partially or completely SERT knockout male rats, respectively.

## Figures and Tables

**Table 1:** Baseline levels of 5-HT and 5-HIAA (nM) in the NAc and mPFC. The groups SERT^−/−^ and SERT^−/+^ were compared to SERT^+/+^. Significance is described as * *p* < 0.05 and ** *p* < 0.01.

Brain Area	Genotype	5-HT (nM)	5-HIAA (nM)
NAc	SERT^+/+^	0.11	154.25
	SERT^+/−^	0.15	122.51 *
	SERT^−/−^	0.64 **	86.375 **
mPFC	SERT^+/+^	0.16	65.67
	SERT^+/−^	0.22	51.34 **
	SERT^−/−^	0.71 **	34.91 **
